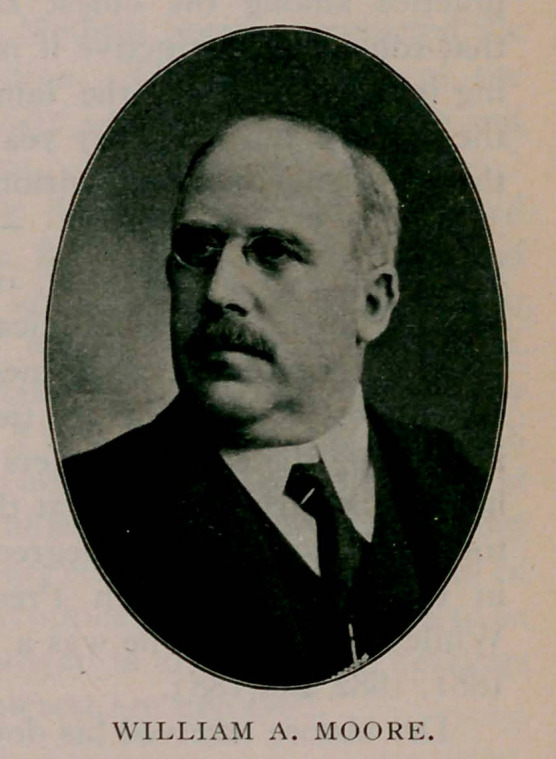# The State Medical Society in 1902

**Published:** 1902-03

**Authors:** 


A Monthly Review of Medicine and Surgery.

*EDITOR:*

WILLIAM WARREN POTTER, M. D.

All communications, whether of a literary or business nature, books for review and exchanges should be addressed to the editor: 284 Franklin Street, Buffalo, N. Y.

Vol. XLI.—LVII. MARCH, 1902. No. 8.

### The State Medical Society in 1902.

THE recent annual meeting—the ninety-sixth—of the Medical Society of the State of New York, was a splendid gathering of representative physicians from all parts of the State. The attendance was unusually large, the total registration being 537, and among the visiting members from other states were men of distinction, who contributed in large measure to the scientific and social features of the meeting.

The president, Dr. Henry L. Elsner, of Syracuse, distinguished his administration by conducting the affairs of the society for a year in such a manner that its traditions for usefulness have been revived, its scientific standing strengthened and its esprit de corps greatly increased. During his year of service he visited many of the county societies throughout the state, reading papers before them and taking an active part in their proceedings. This served to stimulate interest not only in the local bodies, but in the parent or state society, and good results were witnessed at the Albany meeting. It requires a goodly expenditure of time and money to do such splendid work, and the thanks of the profession is due to President Elsner for the liberal policy which he inaugurated in the direction named.

He also organised and conducted a successful semiannual meeting at New York in October, 1901, that was a most profitable innovation which deserves to be repeated. Through his considerate action much has been done in the year to develop a feeling of good fellowship among the profession of the entire state. Members of rival societies have felt and acknowledged this fact and have accorded generous praise for his efforts in this direction. More than all, he has laid the foundation for an ultimate union of the profession in a single state society, which, if not accomplished, will be no fault of his. His suggestion is set forth in his inaugural address as follows:

I recommend that the Medical Society of the State of New York appoint a committee of five, to confer with an equal number representing the New York State Medical Association, for the purpose of formulating a plan which shall have for its object the reorganisation of the regular profession of this state, which body shall be in affiliation with the American Medical Association, and that the committee report the result of its labors at the next meeting of the Medical Society of the State of New York.

In the event of the failure of the New York State Medical Association to appoint such a committee, or if the committees should fail to agree upon a plan of reorganisation, the committee appointed by the Medical Society of the State of New York shall have full power, if it deems it expedient, to represent this society before the American Medical Association, and the secretary of this society shall, if the majority of the committee desires, provide the individual members with credentials of delegates to the American Medical Association.

Surely, no physician can take exception to the plan Dr. Elsner has presented, and it ought to meet with cordial approbation on all sides. The society authorised the appointment of the committee as recommended, providing that the president himself should head it. The committee, as subsequently announced, is as follows: Henry L. Elsner, Abraham Jacobi, Albert Vander Veer, A. M. Phelps and George Ryerson Fowler. The personnel of the committee is such as to justify hopefulness of its success, and if equally prominent and representative men are appointed by the other body it will be a cause for thankfulness.

The program, prepared under the direction of the business committee, of which Dr. Nathan Jacobson was chairman, had been made up with great care and presented a large group of interestingly instructive papers. The symposia upon paresis and diseases of the pancreas deserve special mention for the scientific thought which they both suggested and developed. The president's address, the text of which being The Value to a Physician of Modern Methods of Diagnosis, was a paper of great practical importance as well as scientific worth.

Among the out-of-state visitors were Drs. F. X. Dercum, Chas. P. Noble and Henry W. Cattell, of Philadelphia, Edward Cowles, of Boston, Denslow Lewis, of Chicago, R. H. Chittenden, of New Haven, W. S. Thayer and J. C. Bloodgood, of Baltimore, whose papers were distinctive features of the program.

The social side of the meeting was well sustained, beginning with a dinner to the president and ex-presidents, by Dr. Daniel Lewis, at the Fort Orange Club, on Monday evening, January 27, and continuing on Tuesday and Wednesday with luncheons and dinners, among which those by Dr. Albert Vander Veer, Dr. William C. Krauss, and Mr. William J. Evans were conspicuous, and terminating with a reception by the president and the annual banquet of the society, at the Ten Eyck, Wednesday evening, January 29. The banquet was one of the best the society has ever given. There were 325 chairs taken and after the large banquet hall was filled to overflowing the annex was quickly appropriated and its capacity taxed to its utmost. The music was excellent and the inspiring responses from the floor added picturesqueness to the scene that was enjoyed from the mezzanine by the ladies.

Speeches at the dinner were made by President Elsner, Governor Odell, Dr. A. Jacobi, Dr. W. G. Macdonald and several other post-prandial speakers of distinction. Dr. Arthur G. Root acted as master of ceremonies and conducted them with élan, his witty interlocutory utterances adding much to the zest of the occasion.

---

With all this splendid enthusiasm marking the first two days, the third day, it must be confessed, might and ought to have been better. Some plan should be devised to hold the attention for three full days and thus avoid crowding the program during Tuesday and Wednesday.

---

Dr. Elsner proved a model presiding officer and conducted the work with despatch, never once permitting the business to lag. It is a rare accomplishment.

---

Dr. Lucien Howe, of Buffalo, was awarded the Merit H. Cash prize.

---

The Buffalo and Erie County contingent was made up of the following-named members and delegates: Drs. Henry R. Hopkins, Roswell Park, William Warren Potter, William C. Krauss, Lucien Howe, F. E. Fronczak, Irving M. Snow, J. G. Thompson, Henry Lapp, R. S. Myers, Ernest Wende, Walter D. Greene, Edward Clark, Arthur W. Hurd.

---

The election of officers resulted as follows: President, Henry Reed Hopkins, of Buffalo; vice-president, William A. Moore, of

Binghamton; secretary, Frederic C. Curtis, of Albany; treasurer, O. D. Ball, of Albany.

---

Since the foregoing was reduced to type, the council of the New York State Medical Association has appointed the following-named Committee of Conference: E. Eliot Harris, chairman, Wm. H. Biggam, Emil Mayer, Parker Syms, Frederick Holme Wiggin, and the president, A. A. Hubbell, ex-officio.

---

THE PRESIDENT AND VICE-PRESIDENT ELECT.

Dr. Henry Reed Hopkins, of Buffalo, was elected president of the Medical Society of the State of New York at its 96th annual meeting, at Albany, January 28-30, 1902. In the history of the

HENRY REED HOPKINS.

WILLIAM A. MOORE.

society, which began in 1806, but five Buffalo physicians have preceded Dr. Hopkins in this honor.

Henry R. Hopkins was born at Jamestown in 1844. His preliminary education was received in the public schools and the Jamestown Academy, after which he came to Buffalo and began the study of medicine in May, 1865. He was graduated from the medical department of the University of Buffalo in 1867 and afterward obtained two years' experience in the Washington hospitals. In January, 1869, he returned to Buffalo and began practice with his distinguished uncle, Dr. Gorham F. Pratt.

He is a member of the Medical Society of the County of Erie, of the American Public Health Association, and of the Buffalo Academy of Medicine. Since 1885 he has been a permanent member of the Medical Society of the State of New York, and for the last five years he has been chairman of the standing committee on hygiene. He is a member of the medical staff and attending physician at the Buffalo General Hospital. For a number of years he taught diseases of the nervous system in the University of Buffalo, and now teaches hygiene in that institution. He is a life member of the Buffalo Historical Society and of the Society of Natural Sciences. He was for many years a vestryman of St. Paul's Church, was a member of the National Guard, attaining the rank of Colonel and Surgeon of the eighth division. He enjoys a very large practice among the oldest Buffalo families, and he belongs to that somewhat distinctive if not exclusive set, now again growing into prominence—the family doctors. His active work in the society during many years has received, in his elevation to the presidency, the recognition it deserves.

---

Dr. William A. Moore, of Binghamton, who was elected vice-president of the Medical Society of the State of New York at its recent annual meeting, belongs to one of the oldest families of the southern tier, his great grandfather numbering among the earliest settlers of Broome County. He received his preliminary education at the high school in Binghamton and took his baccalaureate degree at Columbia University in 1882, in the same class with President Nicholas Murray Butler. While in Columbia he was a member of the varsity crews of 1881, 1882 and 1883.

Dr. Moore received his doctorate degree at the College of Physicians and Surgeons, now the medical department of Columbia University, in 1885, served on the house staff of the Presbyterian Hospital at New York from 1885 to 1887, and began the practice of medicine at Binghamton in 1888. He has become one of the best known operating surgeons in his region, and is surgeon to the Lackawanna Railway, to the Binghamton Street Railway system, and Chief Surgeon to the Binghamton City Hospital. He became a permanent member of the society in 1896.

In Dr. Moore's election, which came to him as a surprise, the society has secured to its official counsels for the current year a man of broad culture, liberal training, and ripe experience.

## Figures and Tables

**Figure f1:**
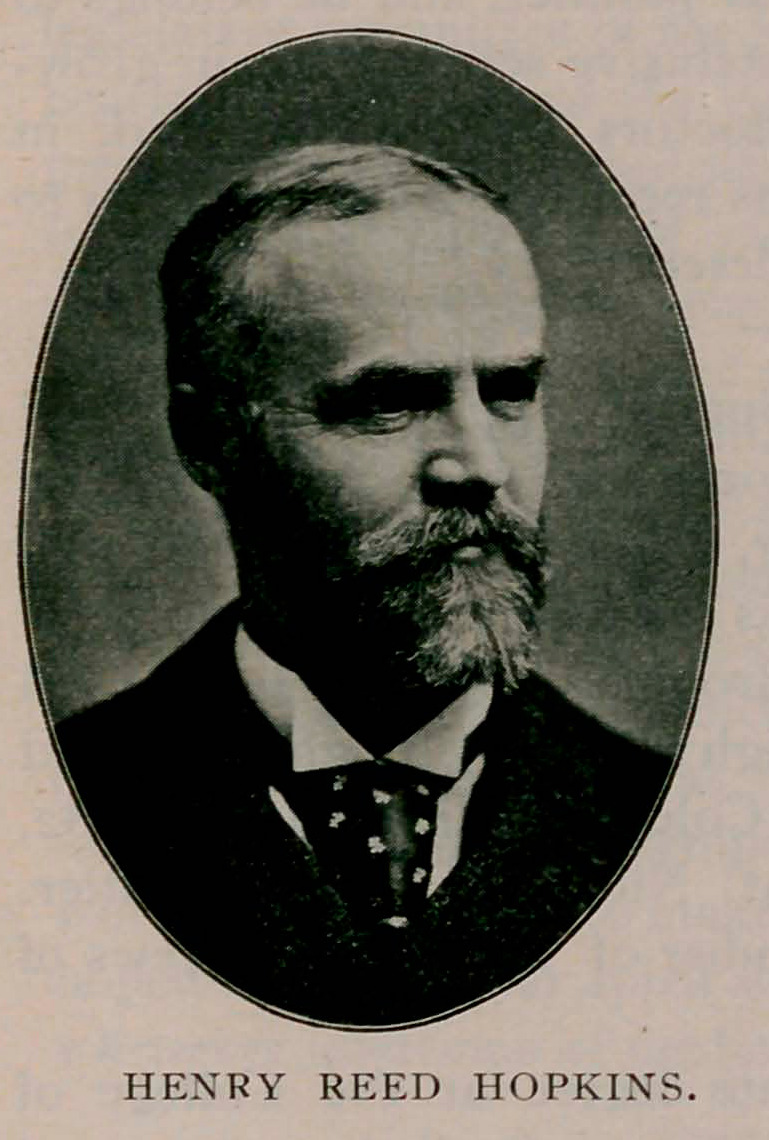


**Figure f2:**